# Network Optimisation and Performance Analysis of a Multistatic Acoustic Navigation Sensor †

**DOI:** 10.3390/s20195718

**Published:** 2020-10-08

**Authors:** Rohan Kapoor, Alessandro Gardi, Roberto Sabatini

**Affiliations:** RMIT University—School of Engineering, Bundoora, VIC 3000, Australia; alessandro.gardi@rmit.edu.au (A.G.); roberto.sabatini@rmit.edu.au (R.S.)

**Keywords:** acoustic, positioning, multistatic, indoor navigation, ultrasonic, autonomous vehicle, unmanned aircraft systems, unmanned ground vehicle, urban canyon

## Abstract

This paper addresses some of the existing research gaps in the practical use of acoustic waves for navigation of autonomous air and surface vehicles. After providing a characterisation of ultrasonic transducers, a multistatic sensor arrangement is discussed, with multiple transmitters broadcasting their respective signals in a round-robin fashion, following a time division multiple access (TDMA) scheme. In particular, an optimisation methodology for the placement of transmitters in a given test volume is presented with the objective of minimizing the position dilution of precision (PDOP) and maximizing the sensor availability. Additionally, the contribution of platform dynamics to positioning error is also analysed in order to support future ground and flight vehicle test activities. Results are presented of both theoretical and experimental data analysis performed to determine the positioning accuracy attainable from the proposed multistatic acoustic navigation sensor. In particular, the ranging errors due to signal delays and attenuation of sound waves in air are analytically derived, and static indoor positioning tests are performed to determine the positioning accuracy attainable with different transmitter–receiver-relative geometries. Additionally, it is shown that the proposed transmitter placement optimisation methodology leads to increased accuracy and better coverage in an indoor environment, where the required position, velocity, and time (PVT) data cannot be delivered by satellite-based navigation systems.

## 1. Introduction

Most human-made navigation systems make use of electromagnetic signals for calculating position, whereas, in nature, mammals like bats use acoustic waves, mostly ultrasound, for navigation and tracking purposes (i.e., echolocation). Acoustic sensors are easy to deploy and have relatively lower cost, size, weight, and power (C-SWaP). Additionally, acoustic navigation is immune to signal-in-space electromagnetic interferences, which affect Global Navigation Satellite System (GNSS) measurements. Since acoustic waves are mechanical waves, their speed varies with changes in medium and environmental conditions. The characterisation of Acoustic Positioning and Navigation System (APNS) includes analysing the linear variation of signal delay at the receiver and angular variation of signal strength, along with error budgeting for the performance of the navigation system. Acoustic sensors, particularly of the ultrasonic type, till now have been widely used for obstacle detection in automobiles, ground robots and small unmanned aerial platforms. However, very limited research has addressed the use of acoustic waves as a means of indoor navigation. As opposed to the extensive application of GNSS outdoors, a low cost, size, weight, and power (C-SWaP) indoor navigation and guidance system is yet to be developed [1]. In the last few decades, the domain of outdoor navigation technologies has witnessed a rapid scale of development, especially with the deployment of satellite-based navigation system constellations. However, people normally spend about two-thirds of their time indoors. Although considerable research has gone into the development of indoor navigation systems, there is still no legacy solution for indoor localisation yet. Current indoor navigation systems require customised local infrastructure based on analysis of requirements for every application [2].

The performance of acoustic navigation systems is not affected by low visibility conditions, such as fog, smoke or lack of illumination, which gives them an advantage over vision-based sensors. The application of acoustic navigation systems to obstacle detection and localisation in unmanned platforms is promising, especially in GNSS (Global Navigation Satellite System)-denied environments, besides being well-suited for path planning in fully autonomous missions. GNSS signals indoors are prone to data degradations or complete loss of signal due to multipath effects, antenna obscuration, or interference. Motion capture systems [3], GNSS repeaters [4], and pseudolites [5] provide accurate positioning indoors, albeit at a higher cost. Various other indoor navigation techniques have been proposed in the literature, including millimetre wave radar [6], using magnetic field data to improve accuracy of inertial measurement units (IMUs) [7], a combination of laser, camera and IMU [8], and radio frequency identification (RFID) [9]. One of the pioneer works conducted in passive acoustic beacons is the “Cricket” indoor location system. This system uses RF to transmit location information, while concurrently transmitting an ultrasonic pulse. Decentralised design of the system enables building-wide deployment. Various interference scenarios involving RF and ultrasonic, both direct and reflected signal, are also investigated [10]. An ultrasonic local positioning system consisting of system-on-chip architecture based on a field-programmable gate array device has been developed for localisation of robot in a complex indoor environment [1]. There are commercially available ultrasonic localisations systems that enable designation of active or passive beacon system, based on user requirements [11,12]. This research aims to characterise acoustic transmission, propagation and reception to optimise the design of an acoustic positioning and navigation system and eventually develop a system that does not rely on RF for synchronisation. Unlike vision-based sensors, acoustic sensors are ideal for navigation applications in low illumination conditions or in the presence of obscurants. Acoustic sensors have a typical advantage over magnetic and tactile sensors due to their robustness to environmental disturbances. Additionally, the ability of acoustic navigation systems to provide sub-centimetre accuracy [11] can be instrumental in safely navigating the unmanned platform through environments ranging from indoor cramped spaces to dense urban environments to caves and mines.

This paper presents the APNS, which uses time division multiple access (TDMA) to obtain a positioning solution of the acoustic receiver in an indoor environment. Previously developed acoustic propagation error models [13] are utilised for designing the optimal transmitter arrangement with the objective of maximizing the coverage of the navigation system and keeping the position dilution of precision (PDOP) values within an acceptable threshold. The main sources of navigation error are introduced along with the discussion of additional positioning error due to platform dynamics in a TDMA navigation system. The results from static positioning tests performed on two different transmitter arrangements are discussed in a comparative analysis. Finally, the paper concludes by summarizing the results and makes recommendations for further work in advancing the research.

## 2. Sound Attenuation

Acoustic waves are transverse mechanical waves that, unlike electromagnetic waves, require a material medium to propagate. The propagation of sound through the air is affected by geometric divergence and atmospheric absorption, with the assumption of a point source of sound. The sound intensity spreads as a spherical wavefront, with the geometric divergence (*A_div_*) solely dependent on the distance from the sound source. The oscillation of air increases with the increase in the frequency of sound as it propagates due to the oscillation of air molecules about their mean position. Frictional losses due to vibration of air molecules and interaction of water vapor with the resonance of oxygen and nitrogen molecules lead to loss of energy. The frequency dependence of the attenuation coefficient for sound in air has three distinct regions due to two relaxation frequencies associated with oxygen and nitrogen.

Sound attenuation is dominated by vibrational relaxation of nitrogen molecules at sound frequencies that are much lower than that associated with oxygen molecules. The apparent bulk viscosity associated with the nitrogen relaxation has a quadratic frequency dependence. The sound frequency is substantially lower than that associated with oxygen relaxation, with quadratic frequency dependence, smaller coefficient and apparent bulk viscosity. On the other hand, the sound frequency is still substantially larger than the frequency associated with nitrogen relaxation in the intermediate region. In the high-frequency region, with an even smaller coefficient, and with the intrinsic bulk viscosity associated with molecular rotation, there is a quadratic frequency dependence [14]. Besides geometric divergence (*A_div_*) and absorption in air (*A_atm_*), the total attenuation of sound in air is also dependent on the surrounding factors like the effect of ground (*A_gr_*) and screening due to an obstacle (*A_bar_*), with the total attenuation of sound in air given by

(1) $$A=A_{div}+A_{\mathrm{atm}}+A_{gr}+A_{bar}+A_{misc}$$

where *A_misc_* is the sound attenuation due to miscellaneous effects such as wind and temperature gradient effects, precipitation, foliage, and housing or industrial sites [15,16]. Additionally, the speed of sound in air also varies with carbon dioxide content (*h_c_*), relative humidity (*h*), temperature (*T*) and barometric pressure. Considering all the above factors, the generalised empirical equation for the speed of sound is given by [17,18]

(2) $$\frac{c}{c_{0}}=a_{0}+a_{1}T+a_{2}T^{2}+a_{3}h_{c}+a_{4}h_{c}T+a_{5}h_{c}T^{2}+a_{6}h+a_{7}hT+a_{8}hT^{2}\;+a_{9}hT^{3}+a_{10}h_{c}^{2}+a_{11}h^{2}+a_{12}hTh_{c}$$

where *c* and *c_0_* are the sound speed and the reference dry-air sound speed, respectively, and *a_0_*–*a_12_* are coefficient constants given in Table 1. Sound speed can be deducted by multiplying Equation (2) by the corresponding reference dry-air sound speed *c_0_*. For a real gas at standard pressure (101.325 kPa), the dry-air sound speed can be approximated to be 331.29 m/s, with an uncertainty of approximately 200 ppm [19], which encompasses sound speeds from 331.224 to 331.356 m/s [20].

## 3. Acoustic Positioning and Navigation System

### 3.1. Ultrasonic Transducer

The ultrasonic transducer is a ceramic microphone that converts electrical energy to mechanical energy, which eventually gets converted to acoustic energy, which is transmitted, while the energy conversion is reversed in the case of the receiver. The transducer has a sandwich construction, with a conical aluminium resonator bonded at the centre of piezoelectric ceramic elements of oppositely polarised material. Because of its shape, the conical resonator begins to vibrate when an ultrasonic signal is applied to the compound vibrator, effectively driving the piezoelectric resonator at its central part. The formation of standing waves inside the case also results in generation of electrical voltage, with the voltage generated from the piezoelectric resonator reaching a maximum when the frequency of the ultrasonic wave applied corresponds to the resonant frequency of the compound resonator. Hence, the resonance of the standing waves formed in the case as well as the compound resonator are considered in the design of an ultrasonic ceramic microphone, thus enabling its function as a high sensitivity transducer.

**Figure 1:**
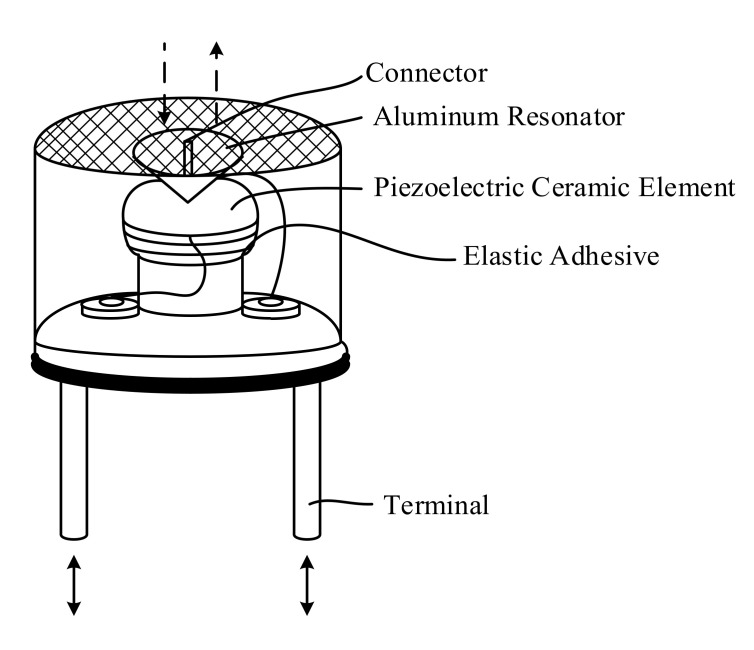
Schematic of an ultrasonic transducer.

The fundamental construction of an open type ultrasonic transducer is shown in Figure 1, with the key specifications listed in Table 2 [21]. The transducer provides a bandwidth of 10 kHz with minimum sensitivity of −70 dB/V/µBar.

### 3.2. APNS Characterisation

**Figure 2:**
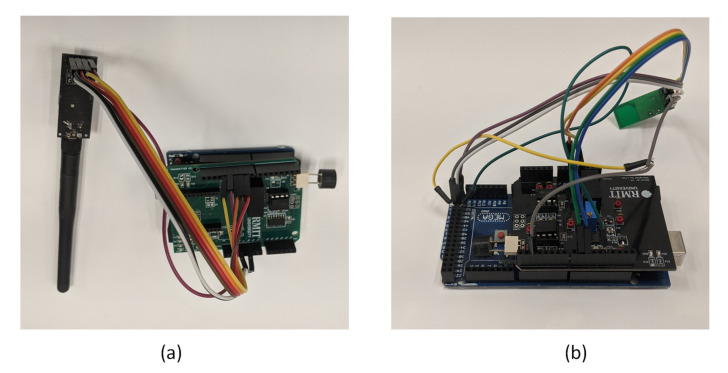
(**a**) Transmitter circuit. (**b**) Receiver circuit.

The transmitter and receiver boards used in the APNS, as shown in Figure 2, were custom developed for conducting the positioning experiments. The boards were designed to fit on Arduino microcontrollers, with provision to function with a standalone power supply. At the transmitter side, a burst of four 40 kHz sinusoidal wave pulses is generated, simultaneously initiating a timer at the receiver. Radio transceivers are currently being employed for clock synchronisation, whereby the time of signal generation at the transmitter is sent to the receiver, and time of flight (ToF) of the acoustic signal is fed to the control station for computation of positioning solution. The transmitter board consists of a microcontroller which generates a 40 kHz wave pulse when triggered by the control station. The ultrasonic pulse is amplified by a differential amplifier, before being transmitted. On the receiver end, there is automatic gain control (AGC) four-stage amplification with a provision for a gain of up to 1000. Because of the requirement for multiple 16-bit timers, Arduino Mega is the microcontroller board used for the receiver circuit. After the received signal is passed through a low pass filter for reduction of noise caused by the power source or other external devices, it is passed through a peak detector with a variable reference voltage. The toggling of the signal, with the detection of a peak at the receiver, terminates the timer, leading to the calculation of ToF of the acoustic signal. Using the speed of sound for the given environmental conditions, the distance from the transmitter to the receiver, or range, is measured. Three or more range measurements are required for the calculation of receiver position using an iterative algorithm. The range measurements are collected at a control station, where post-processing of the data for calculating the receiver position takes place. Further development will enable on-board calculation of positioning solution on the receiver board itself.

Each transmitter emits a burst of four pulses in a fixed round-robin sequence, with the time interval between consecutive transmissions being 30 ms, thus enabling multipath reflections to cease in that time period. Since the linear range of the sensor is about 3 m, most of the reflections from surfaces are considerably weak to be detected at the receiver. All range values greater than twice the maximum range are rejected by the algorithm as well, thus eliminating noise and large multipath errors, if any. Ideally, this allows generation of positioning solution with an update rate of about 8 Hz. However, loss of data packets and noise in the acoustic signal considerably reduces the data update rate. Prior to the improved design, preliminary experiments were carried out on the APNS, results of which can be found in [22]. The new setup is improved to enable wireless synchronisation and data exchange among the mobile receiver and the control station.

## 4. Error Sources in APNS

### 4.1. Propagation Error

The range of acoustic sensors depends on various physical and environmental parameters, as listed in Table 3. The ranging accuracy and resolution determine the performance of the range measurement device. The ranging equation is given by

(3) $$R_{m}=R_{a}+\varepsilon$$

where

(4) $$R_{a}=\sqrt{{\left(x_{T}-x_{R}\right)}^{2}+{\left(y_{T}-y_{R}\right)}^{2}+{\left(z_{T}-z_{R}\right)}^{2}}$$

(5) $$\varepsilon =\varepsilon_{Atm}+\varepsilon_{Ds}+\varepsilon_{Mp}$$

where *R_m_* is the measured range, *R_a_* is the actual range from the transmitter (*x_T_*, *y_T_*, *z_T_*) to the receiver (*x_R_*, *y_R_*, *z_R_*), and *ε* is the error in the measured range. The error term *ε* comprises chiefly of error due to atmospheric effects (*ε_Atm_*), Doppler shift (*ε_Ds_*) and multipath (*ε_Mp_*). The uncertainty in range measurement (*σ_R_*) can be obtained by calculating the deviation in range measurement error from all possible error sources. The cumulative deviation of ranging error is given by

(6) $$\sigma_{R}=\sqrt{\underset{j}{\sum }{\left(\frac{\partial R}{\partial j}\right)}^{2}{\sigma_{j}}^{2}};\;j\in D;\;D=\left\{c_{0},\;t,\;\theta ,\;T_{0},\;\lambda ,\;M,\;c,\;Tr_{i},\;S_{i},\;R_{i},\;v_{w},\;\delta \right\}$$

A case study was conducted to numerically validate the error budget modelling, taking representative numerical values of variables, as shown in Table 4, giving an error of 0.09 m for the indoor test environment conditions. However, in operational situations, the hardware limitations associated with the real-world application of acoustic sensors for navigation can introduce additional errors, which are discussed in the subsequent sections.

### 4.2. Hardware Delays

**Figure 3:**
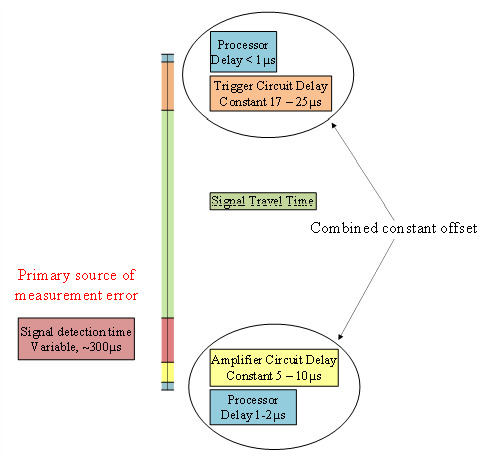
Signal timeline and sources of error (not to scale) [22].

Besides the errors in sound propagation, there are certain hardware limitations at both the transmitter and receiver side which can introduce additional errors due to circuit delays. Most of the circuit delays, as shown in Figure 3, are constant and hence can be removed except the signal detection delays at the receiver, which vary with the distance from the transmitters and have to be experimentally determined in order to be accounted for in the range measurements.

**Figure 4:**
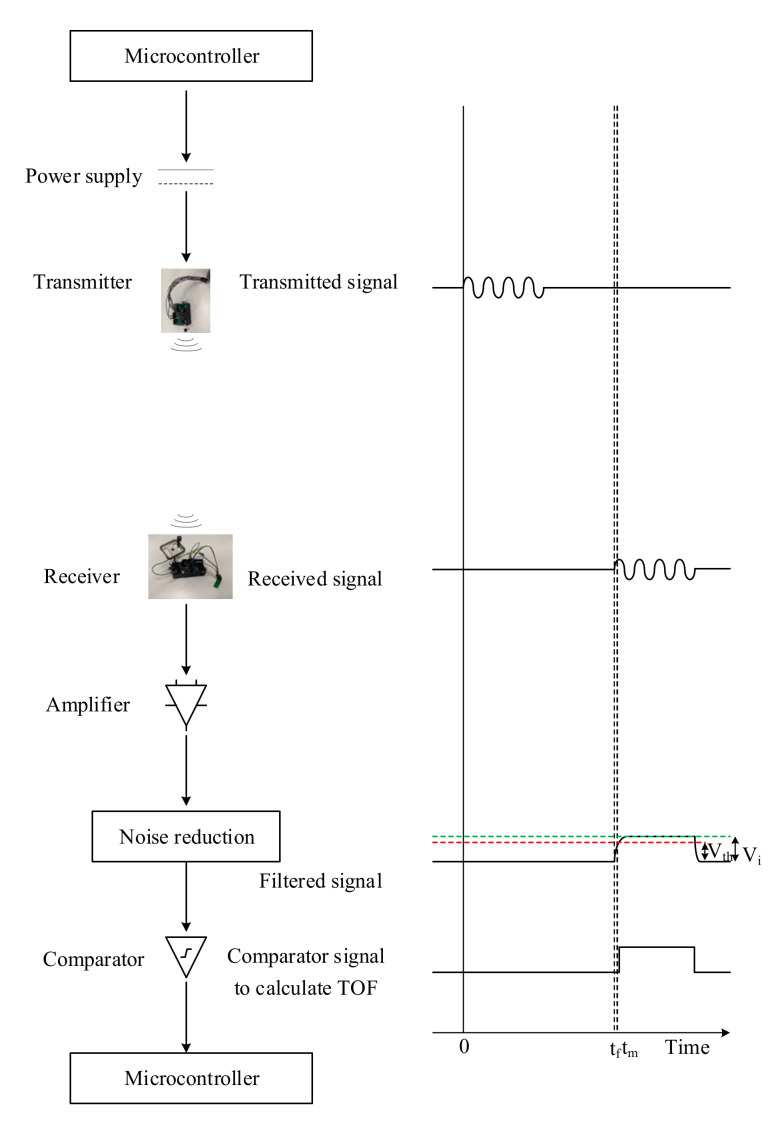
Acoustic positioning and navigation system (adapted from [24]).

The variable delay at the receiver in detecting the incoming acoustic signal is due to the time it takes for the input voltage (*V_i_*) to reach threshold voltage (*V_th_*) [23], as shown in the APNS schematic in Figure 4. The threshold voltage of the comparator circuit (*V_th_*) is related to the input voltage (*V_i_*) by

(7) $$V_{th}=V_{i}\left(1-e^{-\frac{t_{d}}{\tau }}\right)$$

where *τ* is the time constant, and *t_d_* is the time required for the input voltage (*V_i_*) to reach the level of threshold voltage (*V_th_*) of the comparator. Equation (7) can also be written in terms of *t_d_* as

(8) $$t_{d}=-\tau \ln\left(1-\frac{V_{th}}{V_{i}}\right)$$

Denoting other circuit and microcontroller delays at the transmitter and receiver side as *t_o_* and the measured time of arrival (ToA) as *t_m_*, the actual ToA (*t_a_*) can be written as

(9) $$t_{a}=t_{m}-t_{d}-t_{o}$$

The model developed for sound attenuation considers both geometric divergence and atmospheric absorption, while assuming there are no reflections, for the sake of simplification. The input voltage at the receiver (*V_i_*) is inversely proportional to the distance of the receiver to the transmitter, assuming a constant amplifier gain and threshold voltage (*V_th_*). The voltage at the transmitter and the receiver can be assumed to be directly proportional to the sound pressure at the respective ends, while the pressure of a planar sound wave at a distance *r* from a point of pressure *P_0_*, considering the effect of atmospheric absorption, can be calculated as

(10) $$P=\;P_{0}e^{\frac{-\alpha r}{2}}$$

where *α* is the attenuation coefficient for absorption of sound in air, which depends on frequency of sound, humidity, temperature and pressure [13]. Thus, Equation (8) can be written as

(11) $$t_{d}=-\tau \ln\left(1-\frac{V_{th}}{P_{0}e^{\frac{-\alpha r}{2}}}\right)$$

With voltage threshold being constant, *P_0_* can also be assumed to be constant and account for atmospheric absorption. Due to the highly directional nature of the sound waves, the voltage at the receiver due to the transmitted signal depends upon the relative directivity of the transmitter–receiver pair. Denoting the directivity of transmitter and receiver by *k_t_* and *k_r_*, respectively, and inserting the value of *t_d_* in Equation (9), we obtain

(12) $$t_{a}=t_{m}+\tau \ln\left(1-\frac{K}{k_{t}k_{r}e^{\frac{-\alpha r}{2}}}\right)-t_{o}$$

where *K* and *t_o_* are constants and *r* is the distance between the transmitter and the receiver, which can be iteratively calculated as *vt_a_*, based on an initial estimate of *t_a_*. Alternatively, the value of *r* can also be determined independently and put in Equation (12). The constants *K* and *t_o_* can be determined experimentally. The voltage at the receiver is also dependent upon the relative angle (θ) between the transmitter and the receiver. Hence, the transmitter and receiver directivities (*k_t_* and *k_r_*) can be represented by a single function *K*(θ).

**Figure 5:**
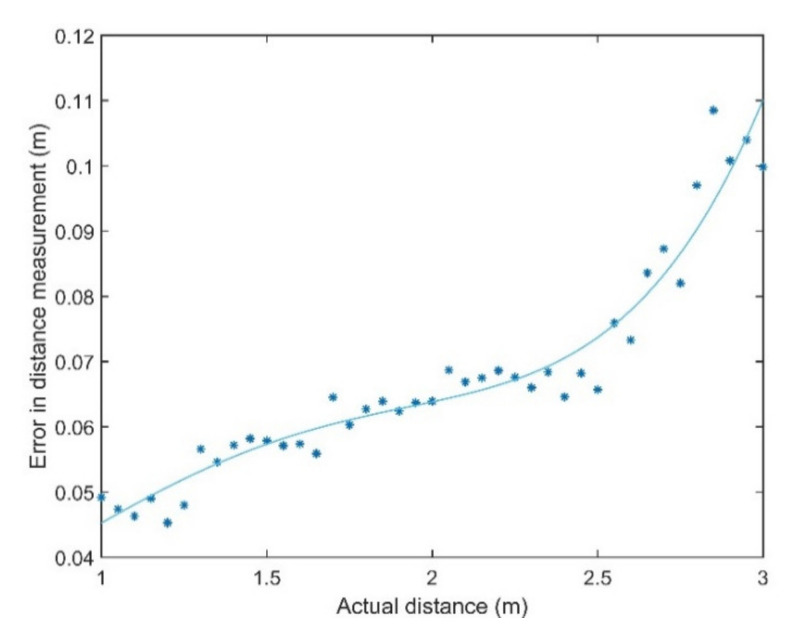
Variation of receiver delay with distance from the transmitter.

However, the variability in signal detection time is mostly due to propagation losses and its uncertainty. Figure 5 shows the variation of delays at the receiver with the distance from the transmitter. It can be observed that there is a sharp increase in receiver delay between the transmitter and the receiver from 2.5 m onwards. A delay of 300 µs can introduce an error of about 10 cm.

### 4.3. Navigation Error

All navigation systems demonstrate a statistical dispersion in their indication of position and velocity, which, as the navigation system becomes more accurate, can be predicted and hence differentiated from the noise [25]. The deterministic errors, like the propagation errors in APNS, are added algebraically, and the statistical errors are root sum squared, while their sum is referred to as total system error.

The mean error and the circular error probability (CEP), also known as the circular probable error (CPE), of the positioning solution are the two parameters that give a measure of the performance of a navigation system. The CEP is the radius of a circle that encloses 50% of the measurements, which in 3D can be represented by the spherical error probable (SEP), which is the radius of a sphere that encloses 50% of all three-dimensional errors. The principal axes are chosen as such that the components of the position errors along each axis are uncorrelated, such that the errors along each axis when plotted separately as cumulative distribution curves, show Gaussianity. One-sigma position errors can also give a measure of the performance of the navigation system. Navigation test data usually contains a bias, leading to non-Gaussian distribution of data, which can be accounted for by taking 95% of the test points centred on the desired navigation fix.

The ranging error is related to the dispersion in measured position by the term geometric dilution of precision (GDOP). If there are three range measurements in orthogonal directions, the standard deviations in position error are the same as those of the three range sensors. However, if the range measurements are more than three or non-orthogonal, the position error can be smaller or much larger than the error in each range measurement. Let $\Delta X={\left[\begin{array}{ccc} \Delta x & \Delta y & \begin{array}{cc} \Delta z & c\Delta t \end{array} \end{array}\right]}^{T}$ be the position and clock offset (Δ*t*) error vector. Let $\vec{\Delta R}={\left[\begin{array}{ccc} \Delta r_{1} & \Delta r_{2} & \begin{array}{cc} \Delta r_{3} & \Delta r_{4} \end{array} \end{array}\right]}^{T}$ be the 1-sigma ranging error vector. Matrix A can be written as

(13) $$A=\left[\begin{array}{cccc} u_{1}^{1} & u_{2}^{1} & u_{3}^{1} & 1\\ u_{1}^{2} & u_{2}^{2} & u_{3}^{2} & 1\\ \vdots & \vdots & \vdots & 1\\ u_{1}^{i} & u_{2}^{i} & u_{3}^{i} & 1 \end{array}\right]$$

where $\left[\begin{array}{ccc} u_{1}^{i} & u_{2}^{i} & u_{3}^{i} \end{array}\right]=\left[\begin{array}{ccc} \cos a_{i} & \cos b_{i} & \cos c_{i} \end{array}\right]$ is the unit vector pointing from the receiver to the *i*^th^ transmitter, with cos *a_i_*, cos *b_i_* and cos *c_i_* being the direction cosines. The ranging error is related to the position and clock offset errors by

(14) $$\vec{\Delta R}=A\Delta X$$

where $\vec{\Delta R}={\left[\Delta r_{1}\Delta r_{2}\ldots \Delta r_{i}\right]}^{T}$ and $\vec{\Delta R}=A\Delta X$. Dilution of precision can be computed from the diagonal elements of ${\left(A^{T}A\right)}^{-1}$, which is the covariance matrix *C*.

(15) $$C={\left(A^{T}A\right)}^{-1}=\left[\begin{array}{cccc} c_{11} & \vdots & \vdots & \vdots \\ \vdots & c_{22} & \vdots & \vdots \\ \vdots & \vdots & c_{33} & \vdots \\ \vdots & \vdots & \vdots & c_{44} \end{array}\right]$$

The PDOP is given by the diagonal elements of the covariance matrix [26]:

(16) $$PDOP=\sqrt{c_{11}+c_{22}+c_{33}}$$

The horizontal dilution of precision (HDOP) and vertical dilution of precision (VDOP) are given by

(17) $$\mathrm{HDOP}=\sqrt{c_{11}+c_{22}}$$

(18) $$VDOP=\sqrt{c_{33}}$$

In pseudoranging systems, the *GDOP* is given by

(19) $${\left(GDOP\right)}^{2}={\left(PDOP\right)}^{2}+{\left(TDOP\right)}^{2}$$

where *TDOP* is the time dilution of precision given by $\sqrt{c_{44}}$. Additionally, *PDOP* can also be represented in terms of residual sum of squares of variances of the position errors or from altitudes of the tetrahedron formed by joining unit vectors from four transmitters and one receiver [27].

(20) $$PDOP=\sqrt{\frac{\sigma_{x}^{2}+\sigma_{y}^{2}+\sigma_{z}^{2}}{\sigma_{R}^{2}}}=\sqrt{\frac{1}{h_{1}^{2}}+\frac{1}{h_{2}^{2}}+\frac{1}{h_{3}^{2}}+\frac{1}{h_{4}^{2}}}$$

where $\sigma_{R}^{2}$ is the variance of range, assuming the value to be equal for each range measurement; $\sigma_{x}^{2}$, $\sigma_{y}^{2}$, and $\sigma_{z}^{2}$ are the variances of the position errors along each axis; and $h_{1}$, $h_{2}$, $h_{3}$, and $h_{4}$ are altitudes of the tetrahedron. The unit vector from a point *R_i_* to a transmitter *T_i_* is given by

(21) $$\hat{RT_{i}}=\frac{\vec{R_{i}}-\vec{T_{i}}}{\left|\vec{R_{i}}-\vec{T_{i}}\right|}$$

**Figure 6:**
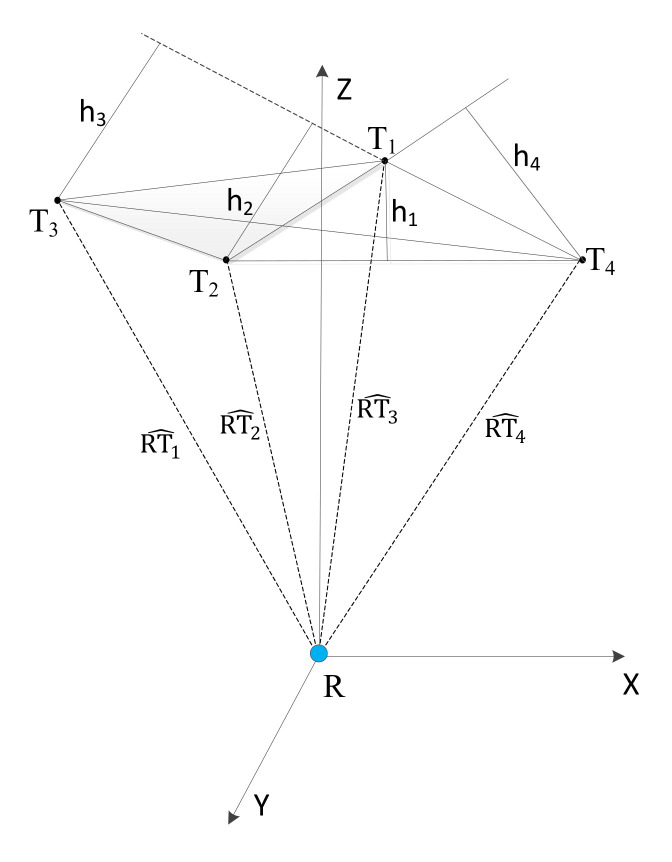
Tetrahedron formed by joining unit vectors from the receiver to the four transmitters.

The tetrahedron formed by joining the unit vectors $\hat{RT_{1}}$, $\hat{RT_{2}}$, $\hat{RT_{3}}$ and $\hat{RT_{4}}$, from the receiver to the four transmitters *T_1_*, *T_2_*, *T_3_* and *T_4_*, as shown in Figure 6, is used for geometrically evaluating the PDOP values. Alternatively, the statistical values of PDOP can also be used to analyse the optimal sensor arrangement [28]. The calculation of statistical values of PDOP requires a priori knowledge of the variances of position errors along each axis as well as the variance in range measurements, which can only be determined experimentally. This paper follows the geometric approach to evaluate the PDOP and performs optimisation of transmitter arrangement for minimizing PDOP in the test volume in the next section, which will subsequently lead to positioning experiments. If there are more than four transmitters, the number of possible combinations (*N*) of four transmitters required to calculate PDOP is given by Equation (22), where *m* is the number of transmitters.

(22) $$N=\frac{m!}{4!\left(m-4\right)}$$

**Figure 7:**
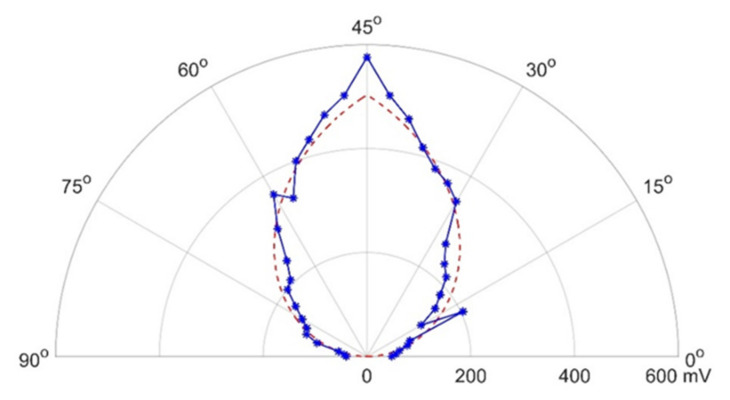
Angular variation of transmitter–receiver directivity.

Figure 7 shows the angular variation of directivity of the transmitter–receiver pair. The optimisation of transmitter deployments, therefore, depends not just on the PDOP values but also on the relative directivity of the transmitter–receiver pair, which determines the transmitter availability [28].

The optimisation of transmitter arrangement can be described by the function defined in Equation (23), where *W_1_* and *W_2_* are the respective weights for *PDOP* and transmitter–receiver directivity function *K(θ)*:

(23) $$f\left(x,y,z\right)=PDOP\cdot W_{1}+\;K\left(\theta \right)\cdot W_{2}$$

The curve fitting equation in Figure 7 is given by

(24) $$voltage=503.5-322.6\times \left|\theta -\frac{\pi }{2}\right|$$

where *θ* is the relative angle between the transmitter and the receiver.

## 5. Results

### 5.1. PDOP Optimisation Case Study

**Figure 8:**
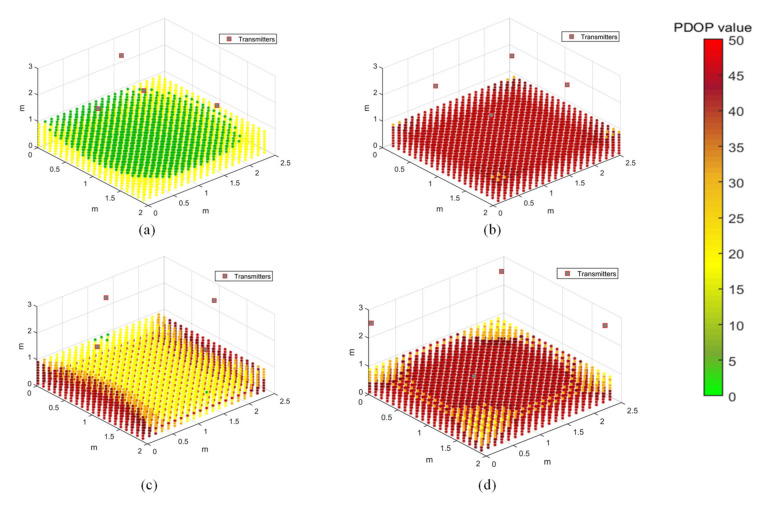
Position dilution of precision (PDOP) distribution for different transmitter arrangements. (**a**) Transmitters arranged in a triangle, with one transmitter at its median. (**b**) Transmitters arranged along a rectangle within the test area. (**c**) Transmitters arranged at the centre of the edges of the test area. (**d**) Transmitters arranged at the corners of the test area.

The PDOP distribution in the test volume is evaluated for four different transmitter arrangements, as shown in Figure 8. This is a limited case of multilateration, with limitations imposed by the hardware limitations of the synchronisation hardware. The transmitters are arranged on the ceiling in a trigonal planar arrangement (Figure 8a), with three transmitters arranged along an equilateral triangle and the fourth transmitter at its median, or in a rectangular pattern which is along the axis (Figure 8b,d) or at the centre of the edges (Figure 8c). A grid of points is evaluated for its PDOP value in a rectangular volume of 2 × 2.5 × 1 m^3^, at about 10 cm increments along the three coordinate axes. The PDOP values can increase significantly in the absence of a transmitter at a higher elevation angle with respect to the receiver. Different transmitter arrangements will be analysed for coverage and accuracy of the positioning solution by conducting positioning tests.

The arrangement of transmitters can significantly impact the accuracy and coverage of the range measurements, thereby affecting the performance of the APNS. The PDOP values provide a measure of the accuracy of the navigation solution at a point, based on the relative geometry of the receiver at that point to the transmitters mounted at known fixed locations. The arrangement of transmitters is optimised with the objective of minimising the PDOP in the test volume using particle swarm optimisation. This optimisation technique finds a local unconstrained minimum to the objective function. After defining the geometric constraints for the test volume, the optimiser calculates the combination of coordinates of the four transmitters which minimise the PDOP throughout the test volume. After defining the bounds, the coordinates of the four transmitters are optimised by evaluating the 12 design variables.

**Figure 9:**
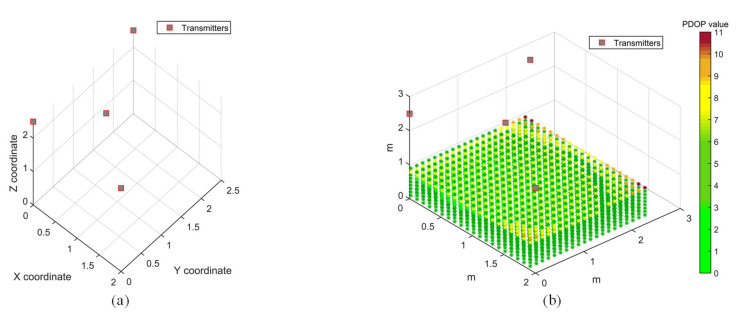
Transmitter arrangement optimisation using PDOP. (**a**) Optimised transmitter arrangement. (**b**) PDOP for an optimised transmitter arrangement.

A test volume of 2 × 2.5 × 2.5 m^3^ was chosen for the PDOP optimisation case study, which is the same as the test volume chosen for PDOP distribution study. The transmitters are constrained by the test volume, and their height is confined to the ceiling. Figure 9a shows the optimised transmitter arrangement, while the PDOP distribution for the optimisation solution is shown in Figure 9b. It can be observed that the PDOP distribution is considerably improved in the test volume as compared to all the cases of PDOP distribution presented in Figure 8. With an optimised arrangement of transmitters, the PDOP value for most of the test volume is below 7. The optimised coordinates for the four transmitters, as shown in Figure 9, are as follows: T_1_ (2,0,2.5), T_2_ (0.79,0.95,2.5), T_3_ (0,0,2.5) and T_4_ (0,2.5,2.5).

However, considering other factors such as range limitations of transmitters and transmitter–receiver directivity constraints, the arrangement shown in Figure 8a, with coordinates T_1_ (0.01,0.05,2.555), T_2_ (1.738,1.243,2.567), T_3_ (0.598,1.222,2.575) and T_4_ (0.035,2.33,2.552), is chosen for conducting positioning experiments. A rectangular arrangement of transmitters is also chosen for positioning experiments to evaluate the effect of transmitter arrangement on positioning accuracy by comparing the two transmitter arrangements.

### 5.2. Receiver Motion Simulation

**Figure 10:**
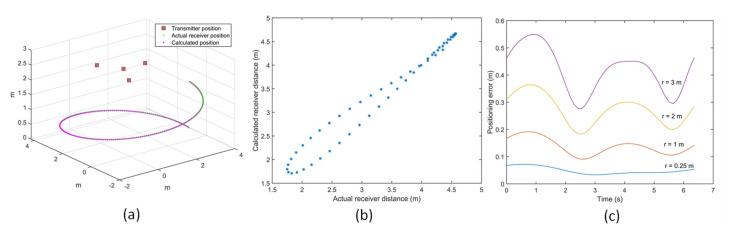
(**a**) Receiver motion in a spiral trajectory at 3 m/s (r = 3 m). (**b**) Actual vs calculated receiver distance at 3 m/s. (**c**) Positioning error propagation due to receiver moving at different velocities.

In addition to propagation errors and error due to hardware delays, the platform dynamics also add to the error in positioning solution, which becomes significant, considering the relatively slow propagation speed of sound. Assuming a time delay of $\Delta_{t}$ between successive range measurements, the error in the range of the $n^{th}$ transmitter ($\delta \vec{R_{i}}$) at a known fixed location to the moving receiver can be written as

(25) $$\delta \vec{R_{i}}=\;\vec{R_{i,M}}-\vec{R_{i}}=\left(n-1\right)\Delta_{t}\;\left(\vec{v}\cdot \frac{\vec{R_{i}}}{\left|\vec{R_{i}}\right|}\right)$$

where $\vec{R_{i,M}}$ and $\vec{R_{i}}$ are the measured and actual ranges, respectively, and the receiver velocity is denoted by $\vec{v}$. Different arrangements of transmitters were tested for the variation of positioning error for the receiver moving at different velocities. Figure 10a shows the error introduced in the receiver coordinates due to receiver motion when the receiver is moving in a spiral trajectory with a constant speed of 3 m/s. Figure 10b shows the comparison of the actual receiver distance with the calculated values. For a fixed angular velocity of 1 rad/s, the receiver is moved in a spiral motion of a varying radius. Figure 10c shows the variation of positioning error for different velocities of the receiver for the chosen optimal transmitter arrangement. For a fixed angular velocity, the variation of positioning error with an increase in velocity of the receiver due to the increase in the radius of the spiral is evaluated. It can be observed that positioning error due to receiver motion increases with the increase in the velocity of the receiver. This is intuitive, as the distance covered by the receiver for a given time delay between successive range measurements increases with the increase in the velocity of the receiver.

The APNS relies on the TDMA approach to calculate the coordinates of the receiver. The receiver motion introduces an error due to a gap of 30 ms between successive range measurements. In a sensor network of four transmitters, it takes about 120 ms to calculate the position of the receiver. In real-life situations, other errors are also introduced due to the Doppler effect and variation of speed of sound due to changes in atmospheric conditions.

Simulations were carried out for evaluating the positioning error due to receiver motion at different time delays between consecutive range measurements using the Monte Carlo method. Four hundred data points were randomly selected for both the receiver’s initial position and its velocity in a given test volume of 2 × 2.5 × 3 m^3^. These points defined the initial position of the receiver as well the receiver velocity, denoted by the length and orientation of the line joining the points. The receiver’s initial position is constrained within the test volume, and the receiver motion is considered in all directions, with the velocity components along each axis being in the range of –5 to 5 m/s in the horizontal direction and –2 to 2 m/s in the vertical direction.

**Figure 11:**
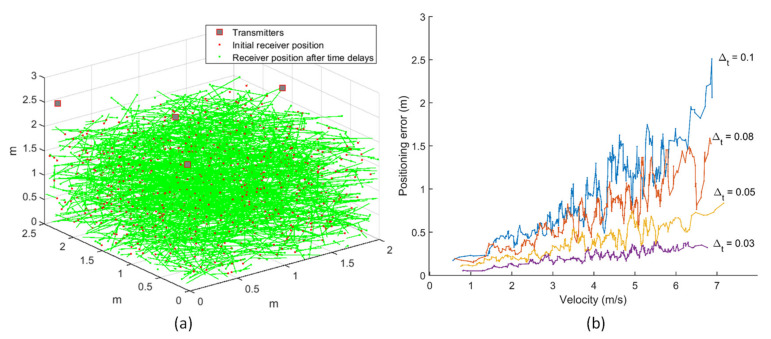
(**a**) Transmitter arrangement and receiver motion. (**b**) Error in positioning due to receiver velocity for various time delays.

The simulations for the optimised transmitter arrangement evaluate the receiver motion in all directions throughout the test volume, as shown in Figure 11a. One set of round-robin range measurements from all four transmitters is represented as a discrete trajectory plot. The variation of positioning error with varying time delays (Δ_t_) is evaluated for receiver motion at different speeds, as shown in Figure 11b. It can be clearly observed that the positioning error increases with the increase in velocity of the receiver. Besides, the magnitude of positioning error for a given velocity also increases with the increase in time delay between successive range measurements. This analysis, along with the dilution of precision (DOP) evaluation, can be employed in system design for optimizing the navigation system performance for a given coverage volume and platform dynamics to deliver the required navigation performance.

### 5.3. Error Budgeting

The error budgeting for conducting positioning tests consists of accounting for range errors in the test volume. Assuming the estimated 1-sigma ranging error (*σ_r_*) to be constant for all range measurements, *σ_r_* combined with the *PDOP* value gives a measure of the estimated positioning error (EPE) or *σ_p_*, given by

(26) $$\sigma_{p}=\sigma_{r}\times PDOP$$

The transmitters were arranged at T_1_ (0.01,0.05,2.555), T_2_ (1.738,1.243,2.567), T_3_ (0.598,1.222,2.575) and T_4_ (0.035,2.33,2.552), in a trigonal planar arrangement. Realistic numerical values of various parameters for calculating the range in an indoor environment were taken, as shown previously in Section 4.1. Compared to the outdoor environment, the horizontal wind velocity is quite low, but the rest of the parameters are of similar value.

The EPE for the positioning tests, discussed in the subsequent section, was calculated to be 1 cm. Another approach using uncertainty analysis for positioning solution, as shown in Equation (27), gave an EPE of 0.8 cm. The latter approach comes in handy when the PDOP values are exceptionally large due to weak transmitter–receiver-relative geometry.

(27) $$\sigma_{p}=\sqrt{{\left(\frac{\partial \Delta r}{\partial x}\right)}^{2}\sigma_{x}{}^{2}+{\left(\frac{\partial \Delta r}{\partial y}\right)}^{2}\sigma_{y}{}^{2}+{\left(\frac{\partial \Delta r}{\partial z}\right)}^{2}\sigma_{z}{}^{2}}$$

However, there are additional errors due to circuit delays which need to be added in the error budget. The total estimated error, including the circuit delay, for static positioning tests is estimated to be 0.9 cm. When conducting flight tests, there are additional errors due to the Doppler effect caused by receiver motion and due to the delay between the consecutive range measurements in the round robin. Additionally, there is a bias in the datum due to synchronisation error between the APNS and datum, which must be adjusted. Table 5 gives the values of 1-sigma estimated errors in the APNS flight tests.

### 5.4. Positioning Tests

**Figure 12:**
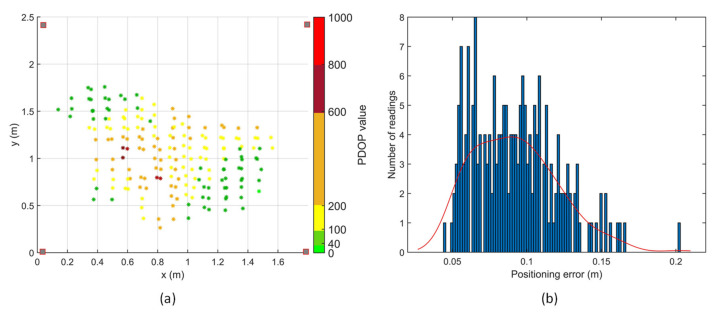
Positioning tests with transmitters arranged in rectangular arrangement.

Positioning tests were performed in two different transmitter arrangements, depicted as Figure 8a,d in the transmitter arrangement optimisation simulations. With an initial estimate, a minimum of three range measurements are required for a positioning solution, which is the intersection of the three spheres with radii equal to the corresponding range measurements. A total of 560 points in the test volume were evaluated for coverage and accuracy of the APNS in the first transmitter arrangement. Out of these, 198 points had 3 or more range measurements, as shown in Figure 12, thus giving a positioning solution. In the second transmitter arrangement, a total of 229 points out of 280 points tested had 3 or more range measurements. Thus, a significantly higher coverage was achieved in the test volume with an optimised arrangement of transmitters.

The accuracy of the APNS was experimentally determined by conducting indoor positioning tests in the given test volume. The positioning results dataset passed the chi-square goodness-of-fit test for normality at a 5% significance level. The PDOP values are considerably poor for the rectangular arrangement of transmitters. Moreover, the coverage of APNS is significantly reduced due to the weak transmitter–receiver-relative geometry. The 1-sigma positioning error for positioning tests conducted with the rectangular arrangement of transmitters is 2.8 cm.

**Figure 13:**
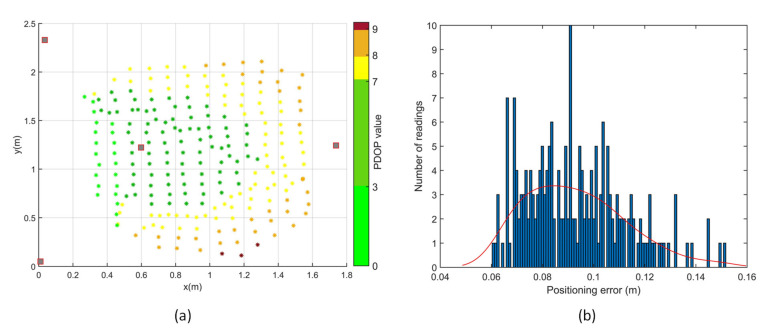
Positioning tests with transmitters arranged in trigonal planar arrangement.

The PDOP values are considerably improved in the trigonal planar arrangement, as shown in Figure 13. The 1-sigma positioning error for positioning tests conducted with the given arrangement of transmitters is 2.0 cm, and the coverage increases by more than double. It can be observed that although the transmitter arrangement does not significantly affect the positioning error, having one transmitter vertically above or at a higher elevation angle with respect to the receiver significantly improves the coverage of transmitters, as shown in Table 6.

**Figure 14:**
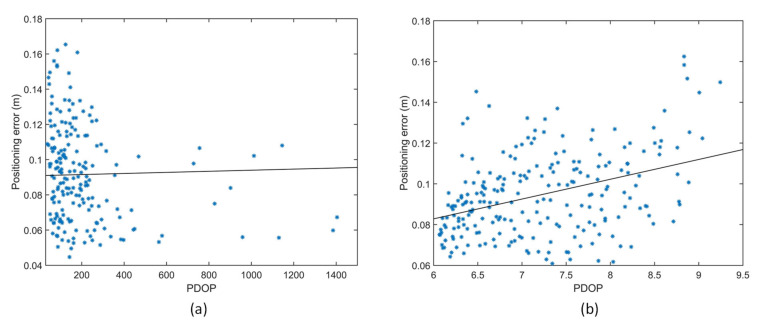
Variation of positioning error with PDOP (**a**) rectangular transmitter arrangement. (**b**) Trigonal planar transmitter arrangement.

Figure 14 shows the variation of positioning error with PDOP in a (a) rectangular and (b) trigonal planar transmitter arrangement. It can be observed that the PDOP has a medium positive correlation with the positioning error in the trigonal planar transmitter arrangement. Thus, an optimised arrangement of transmitters not only leads to higher positioning accuracy due to lower PDOP, but also keeps the cost of the system down by increasing the coverage volume for a given number of transmitters.

## 6. Conclusions

This paper presented the performance modelling and sensor network optimisation methodology for a TDMA-based acoustic positioning and navigation system for indoor and GNSS-challenged environments. An overview of the acoustic navigation system was provided along with the dominating error sources (i.e., propagation, hardware delays and synchronisation) to be accounted for in the overall navigation error budget. Sound wave propagation models were used to account for environmental conditions and the error due to receiver circuit delays were determined experimentally and considered in the navigation solution. Simulation results demonstrated the combined effect of TDMA asynchronous measurements and platform dynamics on the positioning error budget. Particle swarm optimisation techniques were used for calculating the minimum PDOP for transmitter arrangement in the given test volume. The proposed optimisation method leads to better coverage and reduced positioning error. The results of positioning tests conducted on the APNS showed 1-sigma positioning accuracy of 2 cm. Future research will address in more details the effect of transmitter–receiver directivity and optimisation of transmitter arrangement on the coverage and accuracy of APNS for both ground and aerial platform applications. This analysis will inform the design of an improved navigation system based on predicted coverage and accuracy values and the platform-specific integration/operation requirements. Based on these results, future experiments will be performed with aerial and surface autonomous vehicles, both indoors and in urban environments. Besides the current TDMA approach, the potential of using code division multiple access (CDMA) and frequency division multiple access (FDMA) will also be explored. Finally, for the possible integration of APNS on multirotor aerial platforms, the effect of propeller noise on the achievable navigation performance will be investigated with different platform configurations, and dedicated guidance algorithms will be developed to exploit the available position, velocity and time (PVT) data for both mission planning and real-time trajectory optimisation tasks.

## Figures and Tables

**Table 1:** Coefficients for the computation of *c*/*c_0_* [18].

Coefficient Constants	Value	Unit
a_0_	1.000100	-
a_1_	1.8286 × 10^−3^	°C^−^^1^
a_2_	−1.6925 × 10^−^^6^	°C^−^^2^
a_3_	−3.1066 × 10^−^^3^	-
a_4_	−7.9762 × 10^−^^6^	°C^−^^1^
a_5_	3.4000 × 10^−9^	°C^−^^2^
a_6_	8.9180 × 10^−^^4^	-
a_7_	7.7893 × 10^−^^5^	°C^−^^1^
a_8_	1.3795 × 10^−^^6^	°C^−^^2^
a_9_	9.5330 × 10^−^^8^	°C^−^^3^
a_10_	1.2990 × 10^−^^5^	-
a_11_	4.8016 × 10^−^^5^	-
a_12_	−1.4660 × 10^−^^6^	°C^−^^1^

**Table 2:** Ultrasonic transducer specifications.

Parameter	Value	Unit
Operating temperature range	−40 to 85	°C
Capacitance	2550	pF
Sensitivity	−63	dB typically (0 dB = 10 V/Pa)
Directivity	80 (typically)	degrees
Centre frequency	40	kHz
Capacitance tolerance	±20	%

**Table 3:** Ranging parameters [13].

Type	Parameters
Measured observables	Range, velocity, azimuth, and elevation
Environmental parameters	Temperature, wind, humidity, and environmental layout
Design parameters	Transmitted power, carrier frequency, and pulse repetition frequency (PRF)
Performance indicators	Ranging accuracy and resolution

**Table 4:** Values of ranging variables [13].

Variable	Value	Unit
Time of flight (*t*)	0.00879	s
Speed of sound at sea level (*c*_0_)	340.27	m/s
Direction of receiver motion to the LOS (*θ*)	56.31	deg
Mach number for the sound source (*M*)	0.0029	-
Speed of sound emitted by source (*c*) at 20 °C	345	m/s
Variation of temperature with height (λ)	−0.0065	K/m
Distance between *i^th^* transmitter and reflection point ($\left|Tr_{i}-S_{i}\right|$)	9.98	m
Distance between *i^th^* transmitter and receiver ($\left|Tr_{i}-R_{i}\right|$)	10	m
Distance between *i^th^* receiver and reflection point ($\left|R_{i}-S_{i}\right|$)	0.02	m
Sea-level temperature (*T*_0_)	288	K
Horizontal wind velocity (*v_w_*)	2.95	m/s
Angle of wave front normal with the horizontal (*δ*)	60	deg

**Table 5:** Error budgeting.

Error Source	1-Sigma Error (cm)
Estimated positioning error	0.8
Circuit delays	0.1
Doppler effect (velocity 3 m/s)	0.2
Receiver motion (velocity 3 m/s)	8.2
Total estimated error	9.3

**Table 6:** Positioning test results.

Transmitter Arrangement	Mean Positioning Error (cm)	1-sigma Positioning Error (cm)	Maximum PDOP	Minimum PDOP	Coverage (%)
Rectangular	9.2	2.8	>>99	37.67	35.35
Trigonal planar	9.5	2.0	9.24	6.06	81.78
